# Safety, Tolerability, and Pharmacokinetics of Once-Monthly Oral Islatravir: A Phase 2a Study in Participants at Low Risk for Acquiring Human Immunodeficiency Virus Type 1

**DOI:** 10.1093/infdis/jiaf222

**Published:** 2025-05-06

**Authors:** Sharon L Hillier, Linda-Gail Bekker, Sharon A Riddler, Craig W Hendrix, Sharlaa Badal-Faesen, Pippa Macdonald, Gonasagrie Nair, Johannes Lombaard, Yoseph Caraco, Avivit Peer, Munjal Patel, Ryan Vargo, Brenda Homony, Katherine Nedrow, Barbara Evans, Prachi Wickremasingha, Yun-Ping Zhou, Valerie Teal, Peggy Hwang, Ciaran McMullan, Keith D Kaufman, Michael N Robertson, Rebeca M Plank

**Affiliations:** Department of Obstetrics, Gynecology and Reproductive Sciences, Magee-Womens Research Institute and Foundation, Pittsburgh, Pennsylvania, USA; Department of Medicine, University of Pittsburgh, Pittsburgh, Pennsylvania, USA; Department of Medicine, Desmond Tutu HIV Centre, Institute of Infectious Disease and Molecular Medicine, Faculty of Health Sciences, University of Cape Town, Cape Town, South Africa; Department of Medicine, University of Pittsburgh, Pittsburgh, Pennsylvania, USA; Department of Medicine (Clinical Pharmacology), The John Hopkins Hospital, Baltimore, Maryland, USA; Clinical HIV Research Unit, Faculty of Health Sciences, University of the Witwatersrand, Johannesburg, South Africa; Department of Medicine, Desmond Tutu HIV Centre, Institute of Infectious Disease and Molecular Medicine, Faculty of Health Sciences, University of Cape Town, Cape Town, South Africa; Department of Medicine, Desmond Tutu HIV Centre, Institute of Infectious Disease and Molecular Medicine, Faculty of Health Sciences, University of Cape Town, Cape Town, South Africa; Research and Development, Josha Research, Bloemfontein, South Africa; Clinical Pharmacology Unit, Division of Medicine, Hadassah Medical Center, Jerusalem, Israel; Department of Oncology, Rambam Health Care Campus, Haifa, Israel; MRL Research Laboratories, Merck & Co., Inc., Rahway, New Jersey, USA; MRL Research Laboratories, Merck & Co., Inc., Rahway, New Jersey, USA; MRL Research Laboratories, Merck & Co., Inc., Rahway, New Jersey, USA; MRL Research Laboratories, Merck & Co., Inc., Rahway, New Jersey, USA; MRL Research Laboratories, Merck & Co., Inc., Rahway, New Jersey, USA; MRL Research Laboratories, Merck & Co., Inc., Rahway, New Jersey, USA; MRL Research Laboratories, Merck & Co., Inc., Rahway, New Jersey, USA; MRL Research Laboratories, Merck & Co., Inc., Rahway, New Jersey, USA; MRL Research Laboratories, Merck & Co., Inc., Rahway, New Jersey, USA; MRL Research Laboratories, Merck & Co., Inc., Rahway, New Jersey, USA; MRL Research Laboratories, Merck & Co., Inc., Rahway, New Jersey, USA; MRL Research Laboratories, Merck & Co., Inc., Rahway, New Jersey, USA; MRL Research Laboratories, Merck & Co., Inc., Rahway, New Jersey, USA

**Keywords:** preexposure prophylaxis, PrEP, nucleoside reverse transcriptase translocation inhibition, long-acting, islatravir

## Abstract

**Background:**

Islatravir, a nucleoside reverse transcriptase translocation inhibitor, exhibits high potency against human immunodeficiency virus type 1 (HIV-1), with a long intracellular half-life. The safety, tolerability, and pharmacokinetics of once-monthly oral islatravir were evaluated in adults at low risk of acquiring HIV-1.

**Methods:**

In this double-blind, placebo-controlled trial, participants were randomized 2:2:1 to receive 6 once-monthly doses of islatravir 60 mg, islatravir 120 mg, or placebo. Objectives included assessing safety, tolerability, and pharmacokinetic profiles of islatravir in plasma and its active metabolite, islatravir triphosphate (ISL-TP), in peripheral blood mononuclear cells (PBMCs).

**Results:**

Of 242 participants (islatravir 60 mg, n = 97; islatravir 120 mg, n = 97; placebo, n = 48), most were aged ≤45 years (90.1%), female (67.4%), and White (52.9%). Proportions of participants experiencing ≥1 adverse event (AE) were similar in the islatravir (60 mg: 68.0%; 120 mg: 64.9%) and placebo (75.0%) arms. AEs were generally mild to moderate, with infection-related AEs comparable across arms. Lymphocyte count decreased in the islatravir arms, with mean percentage changes of −21.3% ± 20.1% (60 mg) and −35.6% ± 22.8% (120 mg) versus +4.4% ± 25.9% (placebo) at week 24. Median intracellular PBMC ISL-TP concentrations remained above the prespecified pharmacokinetic threshold for HIV-1 prophylaxis (0.050 pmol/10^6^ cells) through 4 weeks after the first dose and ≥8 weeks after the last dose.

**Conclusions:**

Oral islatravir 60 mg and 120 mg once monthly demonstrated similar tolerability and AE profiles to placebo, except for dose-dependent decreases in total lymphocyte counts. A partial recovery in total lymphocyte counts was observed. In most participants, both islatravir doses achieved PBMC ISL-TP exposure levels projected to be effective for once-monthly oral HIV-1 preexposure prophylaxis.

**Clinical Trials Registration:**

NCT04003103.

## Teaser

In this randomized, placebo-controlled trial in adults at low risk of HIV-1 exposure, oral islatravir 60 mg and 120 mg once monthly had a generally favorable adverse event profile, and islatravir triphosphate concentrations in PBMCs were above the prespecified pharmacokinetic HIV-1 preventive threshold.

Innovations in human immunodeficiency virus type 1 (HIV-1) preexposure prophylaxis (PrEP) options are needed to meet the diverse needs of individuals who could benefit from PrEP. Despite efforts to expand HIV treatment and the global recommendation for provision of oral PrEP [1, 2], approximately 1.5 million new HIV infections occurred worldwide in 2022, equating to approximately 4000 people acquiring HIV daily [3].

Current PrEP modalities include daily oral, monthly vaginal ring, and long-acting injectable options [4]. Adherence remains a challenge for daily oral PrEP [5, 6], the vaginal ring is discreet but not widely accessible outside Africa [7–13], and long-acting injectable PrEP requires administration by healthcare providers [14, 15]. Additional options for HIV-1 prevention, including discreet, self-administered, long-acting agents, are needed to overcome barriers associated with low PrEP uptake, adherence, and persistence [16–21].

Nucleoside reverse transcriptase translocation inhibitors (NRTTIs) show promise for treatment and prevention of HIV-1 [22, 23]. Islatravir (ISL), an NRTTI currently being investigated for treatment of HIV-1 [24–26], has high potency, a long half-life (t_1/2_), a favorable drug resistance profile, and broad pharmacologic distribution [27]. The potency of ISL, coupled with the long intracellular t_1/2_ of its active form, islatravir triphosphate (ISL-TP), support its potential as a once-monthly (QM) oral dosing regimen for HIV-1 PrEP.

Based on efficacious concentrations observed in preclinical and early clinical studies [24, 28], a pharmacokinetic (PK) threshold of 0.050 pmol/10^6^ cells for ISL-TP in peripheral blood mononuclear cells (PBMCs) was established for HIV-1 prevention [29, 30]. ISL 60 mg is anticipated to achieve this threshold for ≥4 weeks after dosing, based on PK modeling analyses.

This study evaluated the safety, tolerability, and PK of oral ISL (60 mg and 120 mg) given QM for 6 doses, compared with placebo, in adults at low risk of acquiring HIV-1.

## METHODS

### Study Design and Participants

**Figure 1.**
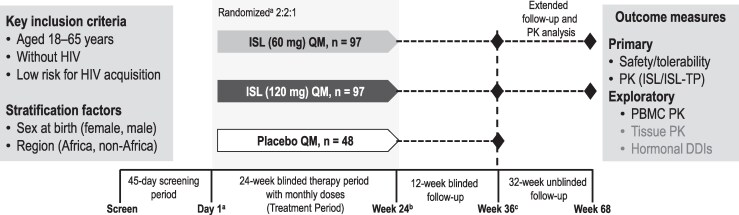
Study schema and treatment plan. ^a^Randomization to study intervention at day 1 and stratified by sex assigned at birth (female, male) and region (Africa, non-Africa). ^b^Sponsor was unblinded at week 24 to allow for an interim evaluation of safety. Participants and investigators/clinical site personnel remained blinded up to week 36. ^c^After week 36, only participants in the peripheral blood mononuclear cell/pharmacokinetic (PK) bridging substudy who were initially randomized to islatravir had an additional 32-week extended, unblinded PK follow-up through week 68. Abbreviations: DDI, drug–drug interaction; HIV, human immunodeficiency virus; ISL, islatravir; ISL-TP, islatravir triphosphate; PBMC, peripheral blood mononuclear cell; PK, pharmacokinetics; QM, once monthly.

This randomized, double-blind, placebo-controlled, parallel-group, multicenter, phase 2a study (MK-8591-016; NCT04003103) evaluated oral ISL QM in adults without HIV and at low risk of acquiring HIV-1. An optional unblinded follow-up phase collected additional PK samples (PBMC/PK bridging subset; Figure 1). The study adhered to Good Clinical Practice principles and received approval from appropriate institutional review boards and regulatory agencies. Written informed consent was obtained before participant screening. Conducted during the coronavirus disease 2019 (COVID-19) pandemic, the study followed health authority guidance from the US Food and Drug Administration and the European Medicines Agency. Measures were implemented to ensure participant and staff safety, maintain Good Clinical Practice compliance, and preserve data integrity. Contingency measures tailored to each country, region, and study site were executed per standard operating procedures for exception and deviation management. All exceptions and deviations were documented.

Participants were enrolled at 9 investigational centers in Israel, South Africa, and the United States. Eligible participants were aged 18–65 years and generally in good health (Supplementary Table 1).

### Study Intervention and Procedures

The study included a screening period (≤45 days), intervention period (24 weeks), 12-week postintervention follow-up period, and an optional 32-week unblinded extended follow-up phase for the collection of ISL PBMC PK samples (the PBMC/PK bridging subset; Figure 1). Sponsor personnel were unblinded at week 24 for interim safety evaluation, while participants and investigators/clinical site personnel remained blinded through week 36 (ie, the 12-week postintervention follow-up period). After week 36, participants and investigators/clinical site personnel were unblinded to study intervention but remained blinded to ISL dose.

Participants were randomized (2:2:1, stratified by sex assigned at birth and region [Africa, non-Africa]) to receive 6 doses of ISL 60 mg, ISL 120 mg, or placebo every 4 weeks for 20 weeks. Participants received an observed oral dose of the assigned blinded study intervention every 4 weeks, starting on day 1, followed by weeks 4, 8, 12, 16, and 20, with the final dosing period through the week 24 visit (including a 14-day window). Participants were followed every 4 weeks for a follow-up period of 12 weeks after the last dosing period (ie, for 12 weeks beyond week 24) through week 36. Participants who discontinued study intervention before week 20 were encouraged to complete all of the follow-up visits.

Adverse events (AEs) were collected at each study visit and were graded according to the Division of AIDS (DAIDS; version 2.1) criteria [31]. Laboratory safety tests included chemistry, hematology, urine point-of-care dipstick, urinalysis, and HIV screening.

Per the original study protocol (19 June 2019), lymphocyte subsets (including CD4^+^ and CD8^+^ T-cell counts) were not collected. In response to findings of decreases in lymphocytes and CD4^+^ T cells in other ISL studies [32], the protocol was amended (8 December 2021) to include additional blood samples for measurement of total lymphocyte, CD4^+^, and CD8^+^ T-cell counts. After study unblinding, lymphocyte data were collected through week 68 (48 weeks after the last dose of drug) in the subset of participants who remained in the trial when these additional laboratory collections were added to the protocol (n = 34). Only serious AEs and nonserious AEs related to study procedures were collected during this extended follow-up.

Median percentage change in weight and metabolic parameters (peripheral fat, trunk fat, and bone mineral density [BMD]) were measured by dual X-ray absorptiometry scans at day 1 and week 24.

Blood samples for determination of levels of ISL in plasma and ISL-TP in PBMC analysis were collected from participants from day 1 through week 24. Predose PK and PBMC samples were collected at visits at day 1 and weeks 4, 8, 12, 16, and 20, with an additional sample taken 30 minutes postdose at day 1, 24 hours after day 1 dose, and week 20. Participants in the PBMC/PK bridging subset who had been randomized to ISL continued into a 32-week unblinded PK follow-up through week 68, with PBMC samples collected at 4 additional visits.

### Outcomes

#### Safety Outcomes

The primary safety outcomes were the incidence of AEs through week 36 and discontinuations due to AEs through week 20. A secondary objective was to evaluate the safety and tolerability of 6 QM doses of ISL (60 mg and 120 mg) through 4 weeks after the last dose (week 24) based on the overall AE profile, including drug-related AEs and serious AEs, and changes in laboratory values.

#### Pharmacokinetic Outcomes

Characterizing the plasma PK profile of ISL was another secondary objective, and characterizing the intracellular PK profile of ISL-TP in PBMCs up to 48 weeks after the last dose of ISL (60 mg and 120 mg) was an exploratory objective of the PBMC/PK substudy. Plasma ISL concentrations and PBMC ISL-TP concentrations were determined by Syneos Health (Quebec, Canada) using a validated high-performance and ultra-performance liquid chromatography–tandem mass spectrometry method, respectively [33, 34]. The analytical range for ISL plasma was 6.82 × 10^−5^ to 6.82 × 10^−1^ µmol/L (0.0682–682.0 nmol/L), with the lower limit of quantitation (LLOQ) at 6.82 × 10^−5^ µmol/L (0.0682 nmol/L). The analytical range for ISL-TP in PBMCs was 1.88 × 10^−4^ to 7.50 × 10^−2^ µmol/L (0.188–75.0 nmol/L), with the LLOQ at 1.88 × 10^−4^ µmol/L (0.188 nmol/L). PBMC cell counts (per 10^6^ cells) were estimated using a hemocytometer, and the conversion from nmol/L to pmol/10^6^ cells used the standard assumption that 1 PBMC has an approximate volume of 0.2 pL [35, 36].

### Statistical Analysis

#### Safety Analysis

All participants receiving ≥1 dose of study intervention were included in the safety population. Point estimates and 95% confidence intervals, for the difference between treatment groups in the proportion of participants with AEs and those who met predefined laboratory changes, were calculated by the Miettinen and Nurminen method. Additional planned safety assessments included mean change in renal parameters (serum creatinine and estimated glomerular filtration rate [eGFR] calculated by the Modification of Diet in Renal Disease equation).

#### Pharmacokinetic Analysis

The plasma PK analysis population included participants treated with ISL who had available data from ≥1 dose of study intervention. Plasma ISL and PBMC ISL-TP concentrations and actual elapsed blood-sampling times relative to ISL time of dose were used to determine the PK profile of plasma ISL and PBMC ISL-TP following doses administered on day 1 and week 20. Plasma ISL and PBMC ISL-TP values below the LLOQ (BLOQ) were treated as 0, except for obvious outliers (eg, BLOQ appeared in the middle of an otherwise nonzero concentration–time profile), which were treated as missing.

The following PK parameter values were determined from concentration–time data by employing a noncompartmental approach using Phoenix WinNonlin software (version 8.1): area under the plasma concentration–time curve from dosing to 672 hours postdose (AUC_0–672h_), maximum concentration (C_max_), time to maximum concentration (T_max_), time to last measurable concentration, trough concentration (C_trough_), and apparent terminal elimination t_1/2_. T_max_ values were obtained directly from the plasma and PBMC concentration–time data. AUCs were calculated using the linear-up/log-down method. The apparent terminal elimination t_1/2_ was calculated as (ln[2]/λz), where λz is the apparent first-order terminal elimination rate constant, calculated from the slope of the linear regression of the terminal log-linear portion of the plasma concentration–time profile.

## RESULTS

### Study Participants

**Figure 2.**
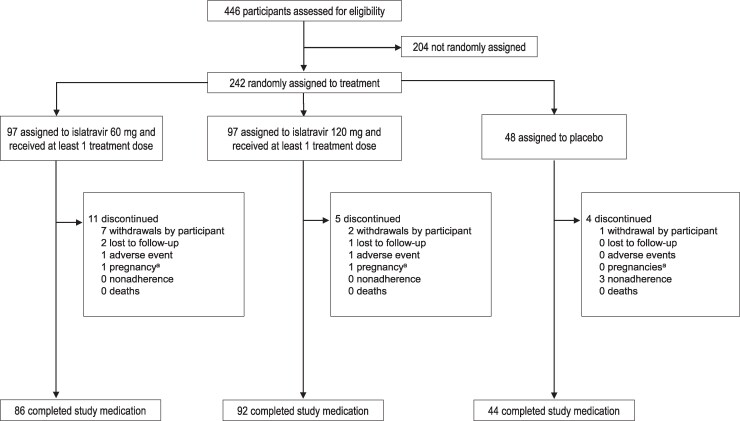
Participant disposition for study medication. ^a^Participants who became pregnant during the study were followed through completion of the infant safety follow-up when the infant reached 1 year of age; these participants did not have a final study status.

In total, 446 participants were screened and 242 were randomized and received ≥1 dose of ISL or placebo. Overall, 91.7% (222/242) of participants completed study medication (Figure 2) and 90.0% (218/242) completed the trial. The plasma PK analysis included 194 participants who had ≥1 dose of ISL (n = 97 each in the ISL 60 mg and 120 mg groups). The PBMC/PK subset included 40 participants who received ISL 60 mg and 41 participants who received ISL 120 mg.

Baseline characteristics were comparable across treatment groups; most participants were aged ≤45 years (90.1%), female (67.4%), and White (52.9%), and the largest proportion of participants were from the United States (45.9%) (Table 1).

**Table 1.** Demographics and Baseline Characteristics

Characteristics	ISL 60 mg (n = 97)	ISL 120 mg (n = 97)	Placebo (n = 48)	Total (N = 242)
Age, y				
Median (range)	32.0 (20–58)	31.0 (18–58)	30.0 (19–45)	31.0 (18–58)
18–45 y	84 (86.6)	86 (88.7)	48 (100.0)	218 (90.1)
Sex assigned at birth				
Female	66 (68.0)	64 (66.0)	33 (68.8)	163 (67.4)
Male	31 (32.0)	33 (34.0)	15 (31.3)	79 (32.6)
Country				
Israel	15 (15.5)	17 (17.5)	13 (27.1)	45 (18.6)
South Africa	34 (35.1)	36 (37.1)	16 (33.3)	86 (35.5)
United States	48 (49.5)	44 (45.4)	19 (39.6)	111 (45.9)
Race				
White	55 (56.7)	44 (45.4)	29 (60.4)	128 (52.9)
Black or African American	37 (38.1)	48 (49.5)	16 (33.3)	101 (41.7)
Other^a^	5 (5.2)	5 (5.2)	3 (6.3)	13 (5.4)

Data are presented as No. (%) unless otherwise indicated. One participant assigned male sex at birth was incorrectly recorded at randomization as female sex assigned at birth.

Abbreviation: ISL, islatravir.

^a^Other race includes Asian, American Indian, Alaska Native, or multiracial.

### Safety

#### Adverse Event Profile

The proportions of participants with ≥1 AE were generally comparable across the treatment groups through week 36: 68.0% (66/97) in the ISL 60 mg group, 64.9% (63/97) in the ISL 120 mg group, and 75.0% (36/48) in the placebo group. AEs resulted in discontinuation of study intervention in 2 participants: 1 from ISL 60 mg discontinued due to rash and pruritus (both grade 2), and 1 from ISL 120 mg discontinued due to foreign body sensation (grade 1, lump in the throat); all 3 events were considered drug-related.

Overall AE profiles were generally comparable across treatment groups through the end of the active treatment period (Table 2), and most AEs were classified as grade 1 or 2. Overall, <5% of participants had grade 3–5 AEs, and no AE was considered drug-related. No clinically meaningful differences between treatment groups were observed for AEs reported by ≥4 participants: the most frequently reported AE was headache in each ISL group, and diarrhea and abdominal pain in the placebo group. Two serious AEs were reported by 1 participant in the ISL 60 mg group during the active treatment period (intentional self-injury and loss of consciousness); neither event was considered drug-related.

**Table 2.** Adverse Event Summary of All Participants as Treated Through the End of the Active Treatment Period^a^

Adverse Event	ISL 60 mg (n = 97)	ISL 120 mg (n = 97)	Placebo (n = 48)	Treatment Difference (95% CI)	
				ISL 60 mg vs Placebo	ISL 120 mg vs Placebo
Participants with ≥1 AE	58 (59.8)	60 (61.9)	32 (66.7)	−6.9 (−22.6 to 10.2)	−4.8 (−20.5 to 12.1)
Toxicity grade 3–5	3 (3.1)	3 (3.1)	1 (2.1)	0	0
Serious AEs	1 (1.0)	0	0	1.0 (−6.5 to 5.6)	0 (−7.5 to 3.8)
Discontinuation due to AE	1 (1.0)	1 (1.0)	0	1.0 (−6.5 to 5.6)	1.0 (−6.5 to 5.6)
Drug-related^b^ AEs	9 (9.3)	14 (14.4)	12 (25.0)	−15.7 (−30.5 to −3.3)	−10.6 (−25.7 to 2.6)
Most common AEs (≥4 participants in any group)					
Headache	10 (10.3)	9 (9.3)	2 (4.2)	6.1 (−4.6 to 14.7)	5.1 (−5.5 to 13.4)
Nausea	5 (5.2)	7 (7.2)	2 (4.2)	1.0 (−9.3 to 8.2)	3.0 (−7.4 to 10.9)
Diarrhea	5 (5.2)	4 (4.1)	4 (8.3)	−3.2 (−14.9 to 5.0)	−4.2 (−15.9 to 3.6)
Vomiting	2 (2.1)	4 (4.1)	1 (2.1)	0 (−9.0 to 5.5)	2.0 (−7.1 to 8.5)
Abdominal pain	3 (3.1)	3 (3.1)	4 (8.3)	−5.2 (−16.8 to 2.2)	−5.2 (−16.8 to 2.2)
Constipation	4 (4.1)	0	2 (4.2)	0 (−10.2 to 6.8)	−4.2 (−14.0 to −0.2)
Upper respiratory tract infection	4 (4.1)	4 (4.1)	3 (6.3)	−2.1 (−13.1 to 5.2)	−2.1 (−13.1 to 5.2)
Blood pressure increased	1 (1.0)	5 (5.2)	0	1.0 (−6.5 5.6)	5.2 (−2.4 to 11.5)
Proteinuria	2 (2.1)	4 (4.1)	1 (2.1)	0.0 (−9.0 to 5.5)	2.0 (−7.1 to 8.5)
Blood creatine phosphokinase increased	1 (1.0)	4 (4.1)	0	1.0 (−6.5 to 5.6)	4.1 (−3.4 to 10.2)

Data are presented as No. (%) unless otherwise indicated.

Abbreviations: AEs, adverse events; CI, confidence interval; ISL, islatravir.

^a^The active treatment period was defined as day 1 through 6 weeks (42 days, based on ISL half-life) after the last dose of study intervention (ie, week 26).

^b^Assessed by the investigator to be related to the drug.

Drug-related AEs were also comparable across treatment groups (Table 3). The most frequent drug-related AEs were headache and pruritus in the ISL 60 mg group, proteinuria in the ISL 120 mg group, and diarrhea in the placebo group.

**Table 3.** Treatment-Related^a^ Adverse Events (Incidence ≥2% in Any Treatment Arm) of All Participants as Treated Through Week 24

Adverse Event	ISL 60 mg (n = 97)	ISL 120 mg (n = 97)	Placebo (n = 48)
Participants with ≥1 drug-related AE	9 (9.3)	14 (14.4)	12 (25.0)
Diarrhea	1 (1.0)	2 (2.1)	4 (8.3)
Proteinuria	1 (1.0)	4 (4.1)	1 (2.1)
Abdominal pain	1 (1.0)	1 (1.0)	3 (6.3)
ALT increased	1 (1.0)	0	3 (6.3)
Nausea	0	2 (2.1)	2 (4.2)
Headache	2 (2.1)	0	1 (2.1)
Pruritus	2 (2.1)	0	1 (2.1)

Data are presented as No. (%). Every participant was counted a single time for each applicable row and column. A system organ class or specific AE was shown only if its incidence in ≥1 treatment group meets the incidence criterion, after rounding.

Abbreviations: AE, adverse events; ALT, alanine aminotransferase; ISL, islatravir.

^a^Assessed by the investigator to be related to the treatment.

During the unblinded follow-up period at week 44, a 29-year-old female in the ISL 120 mg group reported a fetal loss at approximately 11 weeks of gestation. It was estimated that the participant had conceived approximately 10 weeks after receiving the last dose of study medication at week 20; the investigator considered the event unrelated to the study drug. One participant in the ISL 60 mg group died from a cerebrovascular accident at week 64 (44 weeks after receiving the last dose at week 20), which was not considered drug-related by the investigator.

#### Laboratory Tests

There were no clinically meaningful findings in predefined laboratory changes; results were comparable across groups with most treatment-emergent laboratory abnormalities being grade 1 or 2 (Supplementary Table 2). No participant had laboratory values that met criteria for drug-induced liver injury (alanine aminotransferase or aspartate aminotransferase ≥3 × upper limit of normal range [ULN], as well as bilirubin ≥2 × ULN and alkaline phosphatase <2 × ULN). The incidence of treatment-emergent abnormalities in serum creatinine or eGFR was not significantly different between the ISL and placebo groups (Supplementary Table 2). Mean changes from baseline in serum creatinine and eGFR values were small and similar across treatment groups at week 24 (Supplementary Table 3).

At week 24, the median percentage changes from baseline in weight, peripheral fat, and trunk fat were small (<1%) and comparable for the ISL 60 mg and placebo groups (Supplementary Table 3). Participants in the ISL 120 mg group had small increases (2%–3%) in weight, peripheral fat, and trunk fat. Median percentage changes from baseline in total hip BMD and lumbar spine BMD were small and comparable across all groups (Supplementary Table 3).

#### Change in Total Lymphocyte Counts and Other Hematologic Parameters

**Figure 3.**
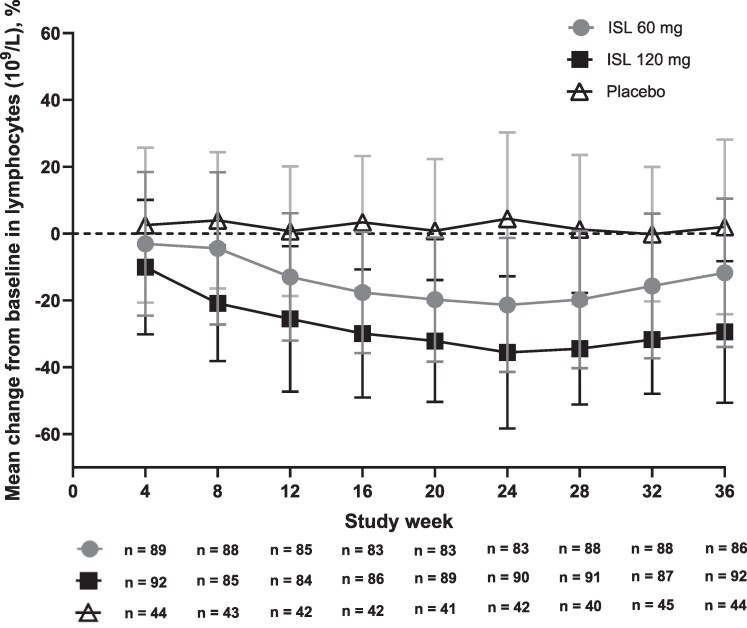
Mean (standard deviation) percentage change from baseline in lymphocyte count over time. Abbreviations: ISL, islatravir; n, number of participants with a test result that met the prespecified criterion.

At week 24, 95% (181/191) of participants in the combined ISL groups had normal total lymphocyte counts (DAIDS grade 0; >650 cells/µL). Of the 10 participants who had lymphocyte decreases that met DAIDS grading criteria (3 in the ISL 60 mg group and 7 in the ISL 120 mg group), 5 had grade 1 (600 to <650 cells/µL), 3 had grade 2 (500 to <600 cells/µL), and 2 had grade 3 (350 to <500 cells/µL) AEs (Supplementary Table 2). None of the participants in the placebo group had lymphocyte decreases that met DAIDS grading criteria. Decreases from baseline in total lymphocyte counts were observed through week 36 for both ISL groups (Figure 3). The largest mean declines were observed at week 24: −21.3% ± 20.1% and −35.6% ± 22.8% for the ISL 60 mg and 120 mg groups, respectively, compared with a 4.4% ± 25.9% increase in the placebo group. By week 36 (ie, 12 weeks after the last dose), the mean lymphocyte declines had improved to −11.8% and −29.4% for the ISL 60 mg and 120 mg groups, respectively, compared with a 2.0% increase in the placebo group (Figure 3).

Lymphocyte data were collected through week 68 (48 weeks after the last dose of study intervention) in the subset of participants who were still in the trial when these additional laboratory collections were added to the protocol. At week 68, mean percentage changes from baseline in lymphocyte count were −9.7% (ISL 60 mg) and −21.5% (ISL 120 mg) (Supplementary Table 4). At weeks 60 and 68, mean CD4^+^ and CD8^+^ T-cell counts in this subset of participants were >600 cells/µL and >300 cells/µL, respectively, for both ISL groups (Supplementary Table 5).

At week 24, no increases in the incidence of infections were observed for participants receiving ISL compared with placebo, or for participants with a decrease in total lymphocyte counts of ≥30% compared with <30% (Supplementary Table 6). No clinically meaningful changes from baseline in other hematologic parameters were observed through weeks 36 and 68.

### Pharmacokinetics

**Figure 4.**
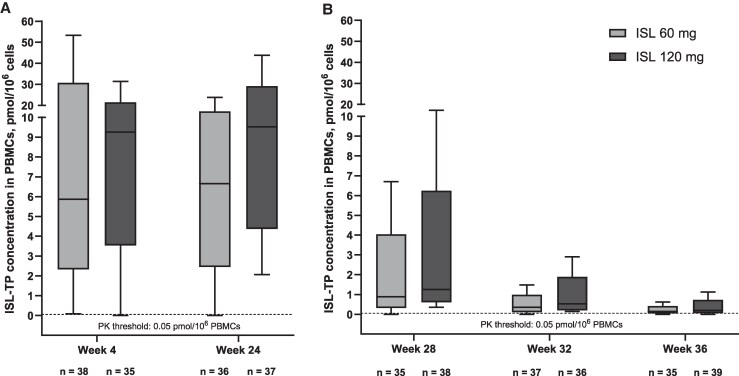
Median (interquartile range) islatravir triphosphate trough concentrations in peripheral blood mononuclear cells at 4 weeks after the first and last dose (*A*) and at 8, 12, and 16 weeks after the last dose (*B*). Abbreviations: ISL, islatravir; ISL-TP, islatravir triphosphate; PBMC, peripheral blood mononuclear cell; PK, pharmacokinetics.

For both ISL doses, biphasic decline was observed in plasma ISL and PBMC ISL-TP levels following peak concentrations; biphasic decline was more pronounced for ISL in plasma compared with ISL-TP in PBMCs (Supplementary Figure 1). The actual times for dosing and PK collection were noticeably different from protocol-scheduled times, which may have contributed to higher values for the geometric mean percent coefficient of variation (Supplementary Table 7). For the ISL 60 mg and 120 mg groups, the geometric mean terminal elimination t_1/2_ of ISL in plasma was 175 and 177 hours, and the t_1/2_ ISL-TP in PBMCs was 483 and 474 hours, respectively (>2.5 weeks; Supplementary Table 7). Median ISL-TP concentrations remained above the prespecified threshold for prophylaxis of 0.050 pmol/10^6^ cells through ≥8 weeks after the last dose (Figure 4). ISL-TP concentrations (C_trough_) remained above the threshold for 94.4% (34/36) of participants 4 weeks after the first 60 mg dose and for 97.1% (34/35) of participants 4 weeks after the last 60 mg dose, with 1 participant's delayed PK collection time significantly later than 672 hours. ISL-TP concentrations remained above the threshold for 91.4% (32/35) of participants 4 weeks after the first 120 mg dose, and for all participants 4 weeks after the last 120 mg dose.

## DISCUSSION

After 6 QM oral doses of ISL 60 mg or ISL 120 mg, adults at low risk for acquiring HIV-1 had an AE profile similar to the placebo group. Safety data through the dosing and follow-up periods were similar across groups, with most AEs categorized as mild or moderate and considered unrelated to the study drug. No drug-related serious AEs were reported. Proteinuria was a frequently reported drug-related AE in the ISL 120 mg group (4.1%, all reported from 1 study site), and there was a high incidence of grade 2 treatment-emergent abnormalities in creatinine clearance across participants, with no significant differences between the ISL and placebo groups. Emergent abnormalities in creatinine clearance were transient and resolved. There were no AEs reported relating to weight, body fat distribution, or BMD in ISL 60 mg. Participants in the ISL 120 mg group had small increases (2%–3%) in weight, peripheral fat, and trunk fat at week 24.

Among participants who received ISL, dose-dependent decreases in total lymphocyte counts were observed, which appeared to be partially reversible during the follow-up period, consistent with results from other studies of ISL as treatment showing a recovery toward baseline after drug discontinuation [32]. Total lymphocyte and subset counts beyond week 36 were available only for a subset of participants; data may not represent the entire study population. Consistent with other studies showing lymphocyte decreases with ISL 0.75 mg once daily or ISL 20 mg once weekly as treatment in people with HIV [32, 37], no increase in infection rates was observed in groups treated with ISL compared with placebo during follow-up [38]. The follow-up period was <1 year, however, which may not be sufficient to fully assess the impact on immunocompetence.

This study reports the longest sampling period for oral ISL-TP to date. The terminal elimination t_1/2_ for oral ISL-TP in PBMCs at 48 weeks after the sixth monthly dose was >470 hours (ie, >2.5 weeks), longer than the 177–209 hours previously reported from studies with shorter PK sampling periods [39, 40]. Effective t_1/2_ based on accumulation ratio was not determined in this study due to considerable deviations in actual PK sample collection, compared with protocol-scheduled timing. These variations resulted from COVID-19–related protocol deviations that permitted flexibility for participants to adjust scheduled study visits. For example, the C_trough_ plasma samples for ISL 60 mg were scheduled to be collected 672 hours after the first and last doses; however, the actual collection times ranged from 459 to 2951 hours after the first dose, and from 408 to 1007 hours after the last dose. Such variations also affected other PK parameters, including concentration in a dosing interval, C_max_, and AUC_0–672_. Additionally, alternate clinical laboratory facilities were allowed for sample collection and processing due to logistical implications and shipping restrictions, impacting sample processing.

This placebo-controlled study allowed for a robust, unbiased evaluation of the tolerability and safety profile of ISL monotherapy in a population of adults at low risk of acquiring HIV-1. Except for a dose-dependent decrease in total lymphocyte counts, ISL was well tolerated at 60 mg and 120 mg QM, with a favorable AE profile. A strength of this study is the enrollment of a diverse population reflecting the population that could most benefit from PrEP: ≥80% of the participants were aged ≤45 years, ≥65% were female, and ≥30% were enrolled in Africa.

Clinical development of oral ISL QM for HIV PrEP has been discontinued due to dose-dependent decreases in total lymphocyte and CD4^+^ T-cell counts seen across the ISL development program [32, 37]. However, ISL continues to be investigated in two 2-drug HIV-1 treatment regimens: an ISL dose of 0.25 mg once daily with doravirine (NCT05631093) and a dose of 2 mg once weekly with lenacapavir (NCT05052996) [38]; the lower dose levels of ISL for treatment have not been associated with decreases in lymphocytes or CD4^+^ T-cell counts. Population pharmacokinetic/pharmacodynamic modeling projects that ISL will achieve efficacious exposures without decreasing lymphocyte and CD4^+^ T-cell counts at these lower dose levels [41, 42].

## CONCLUSIONS

In this randomized, placebo-controlled trial in adults at low risk of acquiring HIV-1, ISL (60 mg and 120 mg) demonstrated tolerability and AE profiles generally comparable to placebo, except for dose-dependent decreases in total lymphocyte counts. Among participants who received ISL, a partial recovery in total lymphocyte counts was observed during the follow-up period in the subset of participants for whom data were available. ISL-TP concentrations for both doses remained above the prespecified PK threshold for prophylaxis of 0.050 pmol/10^6^ PBMCs at the end of the 28-day dosing interval. Although the development of oral ISL QM for HIV-1 PrEP has been terminated due to the concern about lymphocyte toxicity, lower doses of ISL once daily and once weekly continue to be investigated for HIV-1 treatment. The results of this study highlight the potential of other NRTTIs as long-acting oral PrEP for HIV-1, which could contribute to efforts to end the AIDS epidemic by 2030.

## Supplementary Material

jiaf222_Supplementary_Data

## Supplementary Data

Supplementary materials are available at *The Journal of Infectious Diseases* online (http://jid.oxfordjournals.org/). Supplementary materials consist of data provided by the author that are published to benefit the reader. The posted materials are not copyedited. The contents of all supplementary data are the sole responsibility of the authors. Questions or messages regarding errors should be addressed to the author.
