# Supplementary material for: Safety, Tolerability, and Pharmacokinetics of Once-Monthly Oral Islatravir: A Phase 2a Study in Participants at Low Risk for Acquiring Human Immunodeficiency Virus Type 1
Source: J Infect Dis. 2025 May 6;232(5):1050–60. doi: 10.1093/infdis/jiaf222 (PMC12614974; doi:10.1093/infdis/jiaf222)
Supplement: jiaf222_Supplementary_Data [file jiaf222_supplementary_data.pdf]

Supplemental Materials

Supplementary Table 1. Participant Eligibility Criteria

**Inclusion Criteria**	Per the study protocol, a participant was eligible for inclusion in the study if the participant:
Type of Participant and Disease Characteristics	1. Was in good health with laboratory values at screening within the acceptable ranges in the protocol:<br>• Hemoglobin ≥10.5 g/dL for females and ≥11 g/dL for males<br>• Absolute neutrophil count >1000 cells/µL<br>• Platelet count >125,000/µL<br>• Calculated creatinine clearance >90 mL/minute using the Cockcroft-Gault equation<br>• Alanine aminotransferase and aspartate aminotransferase <1.25 x ULN<br>• Total bilirubin ≤ ULN unless history of Gilbert's disease (if Gilbert's disease was the proposed etiology, this was documented in the participant's chart)<br>2. Was confirmed HIV-seronegative based on central laboratory HIV-1/HIV-2 testing results before randomization<br>3. Had a low self-reported risk of acquiring HIV or as per medical history (if available), defined as all of the following within 12 months prior to the screening visit or rescreening visit (if applicable):<br>• No anal or vaginal intercourse with more than 3 partners, or with someone known to be living with HIV, or with someone of unknown HIV serostatus who had an increased risk of acquiring HIV<br>• No stimulant (nonphysician prescribed pharmaceutical-grade stimulants) or illicit drug use of any kind, or inhaled nitrate<br>• No sexually transmitted diseases such as gonorrhea, trichomoniasis, or incident syphilis<br>• No history of antiretroviral use for HIV-1 treatment, PrEP, or post-exposure prophylaxis
Demographics	4. Had provided documented informed consent for the study, including for future biomedical research (by the participant or legally acceptable representative)<br>5. Was male or female at birth, from 18 years to 65 years of age inclusive, at the time of signing the informed consent
Contraception/Pregnancy	6. Was on contraceptives of any kind that were consistent with local regulations regarding the methods of contraception for those participating in clinical studies

	7. Was not a person of childbearing potential, or was nonpregnant with a negative, highly sensitive serum pregnancy test (as required by local regulations) within 24 hours before the first dose of study and not lactating and using protocol-approved contraception
**Exclusion Criteria**	Per the study protocol, a participant was excluded from the study if the participant:
Medical Conditions	1. Had hypersensitivity or other contraindication to any of the components of the study interventions as determined by the investigator<br>2. Had an active diagnosis of hepatitis due to any cause, including active HBV (defined as HBsAg-positive) or HCV (defined as detectable HCV RNA)<br>*Note: Past HBV infection or previous HBV vaccination (defined as HBsAg-negative and positive for antibody against HBsAg), or prior/inactive HCV infection (defined as undetectable HCV RNA) were not exclusionary*<br>3. Had a history of malignancy ≤5 years prior to signing informed consent except for adequately treated basal cell or squamous cell skin cancer or in situ cervical cancer<br>4. Had a history or evidence of any condition, therapy, laboratory abnormality, or other circumstance (including drug or alcohol use or dependence) that might, in the opinion of the investigator, confound the results of the study or interfere with participation for the full duration of the study, such that it is not in the best interest of the participant to participate
Prior/Concomitant Therapy	5. Was taking, or anticipated to require, any of the below prohibited therapies from 30 days prior to day 1 through the duration of the study:<br>• Investigational agents/devices, except for study intervention(s)<br>*Note: Participants were not permitted to enroll in a study of an investigational compound or investigational device during the entire study duration. Participants who discontinued study intervention early were required to refrain from enrolling in a study of an investigational compound or investigational device until at least 16 weeks from the last dose of study intervention*<br>• Immune therapy agents, immune modulators, or other immunosuppressive therapies<br>*Note: Time-limited courses of corticosteroids (eg, for asthma exacerbation) were allowed*
Prior/Concurrent Clinical Study Experience	15. Was participating in or had participated in an interventional clinical study with an investigational compound/device within 30 days prior to day 1 through the duration of the study<br>16. Had previously been randomized in a study and received ISL
Diagnostic Assessments	16. Had a QTc interval (using Fridericia correction) ≥460 msec (for males) or ≥470 msec (for females) or has ECG findings deemed abnormal with clinical significance by the investigator or designee at screening
Other Exclusions	18. Was expecting to conceive or donate eggs at any time during the study

	19. Was or had an immediate family member (eg, spouse, parent/legal guardian, sibling, or child) who was investigational site or Sponsor staff directly involved with the study

Abbreviations: ECG, electrocardiogram; HBsAG, hepatitis B surface antigen; HBV, hepatitis B virus; HCV, hepatitis C virus; ISL, islatravir; PrEP, pre-exposure prophylaxis; QTc, corrected QT interval; ULN, upper limit of normal range.

Supplementary Table 2. Proportion of Participants Who Met the Predefined Laboratory Changes (Including Blood Chemistry and Hematology Parameters) Through the End of the Active Treatment Period

Laboratory Finding Criterion	ISL 60 mg n = 97	ISL 120 mg n = 97	Placebo n = 48	Treatment Difference, % (95% CI)[b]	
	Participants, % (n/m)[a]			ISL 60 mg vs placebo	ISL 120 mg vs placebo
**Chemistry**					
**Alkaline phosphatase, IU/L**					
Grade 1: 1.25–<2.5 × ULN	2.1 (2/97)	0	0	2.1 (–5.6 to 7.2)	0 (–7.6 to 3.8)
**Alanine aminotransferase, IU/L**					
Grade 1: 1.25–<2.5 × ULN	4.1 (4/97)	4.1 (4/97)	8.5 (4/47)	–4.4 (–16.2 to 3.5)	–4.4 (–16.2 to 3.5)
Grade 2: 2.5–<5.0 × ULN	1.0 (1/97)	2.1 (2/97)	2.1 (1/47)	–1.1 (–10.2 to 3.8)	–0.1 (–9.2 to 5.5)
**Amylase, IU/L**					
Grade 1: 1.1–<1.5 × ULN	10.3 (10/97)	7.2 (7/97)	6.4 (3/47)	3.9 (–7.8 to 13.0)	0.8 (–10.6 to 9.2)
Grade 2: 1.5–<3.0 × ULN	1.0 (1/97)	8.2 (8/97)	0	1.0 (–6.6 to 5.6)	8.2 (0.4 to 15.5)
**Aspartate aminotransferase, IU/L**					
Grade 1: 1.25–<2.5 × ULN	3.1 (3/97)	7.2 (7/97)	2.1 (1/47)	1.0 (–8.3 to 7.0)	5.1 (–4.5 to 12.5)
Grade 3: 5.0–<10.0 × ULN	0	1.0 (1.97)	0	0.0 (–7.6 to 3.8)	1.0 (–6.6 to 5.6)
**Bilirubin, mg/dL**					
Grade 1: 1.1–<1.6 × ULN	3.1 (3/97)	1.0 (1/97)	0	3.1 (–4.6 to 8.7)	1.0 (–6.6 to 5.6)
Grade 2: 1.6–<2.6 × ULN	0	1.0 (1/97)	0	0.0 (–7.6 to 3.8)	1.0 (–6.6 to 5.6)
**Creatine kinase, IU/L**					
Grade 1: 3.0–<6.0 × ULN	10.3 (10/97)	9.3 (9/97)	6.4 (3/47)	3.9 (–7.8 to 13.0)	2.9 (–8.7 to 11.7)

Laboratory Finding Criterion	ISL 60 mg n = 97	ISL 120 mg n = 97	Placebo n = 48	Treatment Difference, % (95% CI)[b]	
	Participants, % (n/m)[a]			ISL 60 mg vs placebo	ISL 120 mg vs placebo
Grade 2: 6.0–<10.0 × ULN	0	5.2 (5/97)	2.1 (1/47)	–2.1 (–11.2 to 1.8)	3.0 (–6.4 to 9.8)
Grade 3: 10.0–<20.0 × ULN	0	2.1 (2/97)	0	0.0 (–7.6 to 3.8)	2.1 (–5.6 to 7.2)
Grade 4: ≥20.0 × ULN	0	1.0 (1/97)	0	0.0 (–7.6 to 3.8)	1.0 (–6.6 to 5.6)
**Creatinine, mg/dL**					
Grade 2: >1.3–1.8 × ULN or increase to 1.3 to <1.5 × baseline	13.4 (13/97)	12.4 (12/97)	25.5 (12/47)	–12.1 (–27.4 to 1.0)	–13.2 (–28.3 to –0.1)
Grade 3: >1.8–<3.5 × ULN or increase to 1.5 to <2.0 × baseline	3.1 (3/97)	4.1 (4/97)	2.1 (1/47)	1.0 (–8.3 to 7.0)	2.0 (–7.3 to 8.4)
Grade 4: ≥3.5 × ULN or increase of ≥2.0 × baseline	1.0 (1/97)	0	0	1.0 (–6.6 to 5.6)	0.0 (–7.6 to 3.8)
**Estimated creatinine clearance[c], mL/min**					
Grade 2: <90–60 or <30% decrease from baseline	62.9 (61/97)	64.9 (63/97)	66.0 (31/47)	–3.1 (–18.9 to 14.0)	–1.0 (–16.8 to 16.0)
Grade 3: <60–30 or 30–<50% decrease from baseline	3.1 (3/97)	6.2 (6/97)	8.5 (4/47)	–5.4 (–17.2 to 2.1)	–2.3 (–14.4 to 6.2)
Grade 4: <30 or ≥50% decrease from baseline	1.0 (1/97)	0	0	1.0 (–6.6 to 5.6)	0.0 (–7.6 to 3.8)
**Lipase, IU/L**					
Grade 1: 1.1–<1.5 × ULN	6.2 (6/97)	6.2 (6/97)	2.1 (1/47)	4.1 (–5.4 to 11.2)	4.1 (–5.4 to 11.2)
Grade 2: 1.5–<3.0 × ULN	4.1 (4/97)	5.2 (5/97)	2.1 (1/47)	2.0 (–7.3 to 8.4)	3.0 (–6.4 to 9.8)
Grade 3: 3.0–<5.0 × ULN	0	3.1 (3/97)	2.1 (1/47)	–2.1 (–11.2 to 1.8)	1.0 (–8.3 to 7.0)
Grade 4: ≥5.0 × ULN	1.0 (1/97)	0	0	1.0 (–6.6 to 5.6)	0.0 (–7.6 to 3.8)

Laboratory Finding Criterion	ISL 60 mg n = 97	ISL 120 mg n = 97	Placebo n = 48	Treatment Difference, % (95% CI)[b]	
	Participants, % (n/m)[a]			ISL 60 mg vs placebo	ISL 120 mg vs placebo
**eGFR[d], mL/min/1.73m²**					
Grade 2: <90–60 or 10–<30% decrease from baseline	58.8 (57/97)	62.9 (61/97)	57.4 (27/47)	1.3 (–15.4 to 18.5)	5.4 (–11.2 to 22.5)
Grade 3:<60–30 or 30–<50% decrease from baseline	9.3 (9/97)	8.2 (8/97)	14.9 (7/47)	–5.6 (–19.3 to 5.0)	–6.6 (–20.3 to 3.8)
Grade 4: <30 or ≥50% decrease from baseline	1.0 (1/97)	1.0 (1/97)	0	1.0 (–6.6 to 5.6)	1.0 (–6.6 to 5.6)
**Hematology**					
**Hemoglobin, g/dL**					
Grade 1: Male: 10.0–10.9; Female: 9.5–10.4	1.1 (1/95)	3.1 (3/96)	2.2 (1/46)	1.1 (–10.4 to 3.9)	1.1 (–8.5 to 7.1)
**Leukocytes, 10^9 cells/L**					
Grade 1: 2.000–2.499	1.1 (1/95)	5.2 (5/96)	0	1.1 (–6.7 to 5.8)	5.2 (–2.7 to 11.6)
Grade 2: 1.500–1.999	1.1 (1/95)	1.0 (1/96)	0	1.1 (–6.7 to 5.8)	1.0 (–6.7 to 5.7)
**Lymphocytes, 10^9 cells/L**					
Grade 1: 0.600–<0.650	1.1 (1/95)	4.2 (4/96)	0	1.1 (–6.7 to 5.8)	4.2 (–3.7 to 10.3)
Grade 2: 0.500–<0.600	2.1 (2/95)	1.0 (1/96)	0	2.1 (–5.7 to 7.4)	1.0 (–6.7 to 5.7)
Grade 3: 0.350–<0.500	0	2.1 (2/96)	0	0.0 (–7.8 to 3.9)	2.1 (–5.7 to 7.3)
**Neutrophils, 10^9 cells/L**					
Grade 1: 0.800–1.000	0	3.1 (3/96)	0	0.0 (–7.8 to 3.9)	3.1 (–4.7 to 8.8)
Grade 2: 0.600–0.799	1.1 (1/95)	1.0 (1/96)	0	1.1 (–6.7 to 5.8)	1.0 (–6.7 to 5.7)

Laboratory Finding Criterion	ISL 60 mg<br>n = 97	ISL 120 mg<br>n = 97	Placebo<br>n = 48	Treatment Difference, % (95% CI)[b]	
	Participants, % (n/m)[a]			ISL 60 mg vs placebo	ISL 120 mg vs placebo
Grade 3: 0.400–0.599	1.1 (1/95)	0	0	1.1 (–6.7 to 5.8)	0.0 (–7.8 to 3.9)
Grade 4: <0.400	0	1.0 (1/96)	0	0.0 (–7.8 to 3.9)	1.0 (–6.7 to 5.7)
**Platelets, 10^9 cells/L**					
Grade 1: 100–<125	0	1.0 (1/96)	0	0.0 (–7.8 to 3.9)	1.0 (–6.7 to 5.7)

Abbreviations: eGFR, estimated glomerular filtration rate; ISL, islatravir; ULN, upper limit of normal range.

[a]Only participants with on-treatment post-baseline test results that met the predetermined criterion and are worse in grade than at baseline are included; the baseline value is the last value prior to the start of study medication, post-baseline includes data through 42 days after the last dose of study medication.

[b]The 95% CIs for the treatment differences in percent with predefined limits of change in laboratory parameters were calculated using the Miettinen and Nurminen method.

[c]Decreases in eGFR were transient and most met DAIDS laboratory grading criterion based on percent change from baseline rather than absolute values.

[d]Calculated by the modification of diet in renal disease equation.

Supplementary Table 3. Change From Baseline in Renal and Metabolic Parameters of All Participants As Treated at Week 24

	ISL 60 mg (n = 97)	N	ISL 120 mg (n = 97)	N	Placebo (n = 48)	N
**Renal parameters, mean change[a] from baseline, % (SD)**						
Serum creatinine (mg/dL)	0.003 (0.098)	84	0.004 (0.094)	92	0.032 (0.101)	44
eGFR[b] (mL/min/1.73m^2)	0.327 (21.26)	84	–0.686 (21.73)	92	–6.264 (21.30)	44
**Metabolic parameters, median change from baseline, % (IQR)**						
Weight (kg)	0.39 (–2.13 to 3.55)	83	1.82 (–0.93 to 4.40)	92	0.24 (–1.47 to 2.84)	44
Peripheral fat[c] (g)	–0.35 (–5.68 to 6.07)	83	2.50 (–2.80 to 8.10)	89	0.77 (–2.35 to 5.15)	41
Trunk fat[c] (g)	0.95 (–7.74 to 9.17)	83	3.42 (–1.40 to 10.47)	89	0.25 (–7.21 to 4.78)	41
Total hip BMD[c] (g/cm^2)	0.22 (–0.90 to 1.32)	83	–0.09 (–1.19 to 1.30)	87	0.10 (–1.64 to 1.88)	42
Lumbar spine BMD[c] (g/cm^2)	0.53 (–1.37 to 1.55)	82	0.00 (–1.38 to 1.26)	89	0.63 (–1.39 to 1.31)	42

Abbreviations: BMD, bone mineral density; eGFR, estimated glomerular filtration rate; IQR, interquartile range; ISL, islatravir; RBP, retinol-binding protein.

[a]Mean change from baseline is based on the measurements of participants with values at baseline and at the time point assessed.

[b]Calculated by the modification of diet in renal disease equation.

[c]Measured using dual X-ray absorptiometry.

Supplementary Table 4. Percent Change From Baseline in Lymphocytes (10^9/L) of Available Participants As Treated Through Week 68

Visit Week	Mean Change From Baseline (SD), %		Change From Baseline Category, % (n/m)			
			ISL 60 mg		ISL 120 mg	
	ISL 60 mg	ISL 120 mg	≥30% Reduction	<30% Reduction	≥30% Reduction	<30% Reduction
Week 52	−12.59 (26.74)	−21.18 (20.89)	25.0 (2/8)	75.0 (6/8)	36.4 (4/11)	63.6 (7/11)
m	8	11				
Week 60	−9.95 (27.21)	−36.09 (13.62)	36.4 (4/11)	63.6 (7/11)	66.7 (6/9)	33.3 (3/9)
m	11	9				
Week 68	−9.66 (27.64)	−21.45 (17.07)	23.5 (4/17)	76.5 (13/17)	17.6 (3/17)	82.4 (14/17)
m	17	17				

Abbreviations: ISL, islatravir.

m, number of participants with available data at baseline and the post-baseline of interest; n, number of participants with a test result that met the specified criterion.

Supplementary Table 5. CD4+ and CD8+ T-Cell Counts of Available Participants As Treated at Weeks 60 and 68

Visit Week	ISL 60 mg			ISL 120 mg		
	**n**	**Mean (SD)**	**Median (min–max)**	**n**	**Mean (SD)**	**Median (min–max)**
**CD4+ T-cell counts, cells/μL**						
Week 60	7	754.3 (125.52)	795.0 (569–887)	8	722.1 (233.00)	689.0 (375–1149)
Week 68	17	741.4 (209.37)	660.0 (456–1156)	16	729.5 (210.22)	748.5 (387–1161)
**CD8+ T-cell counts, cells/μL**						
Week 60	6	480.8 (206.03)	433.5 (313–878)	7	368.1 (117.94)	356.0 (249–560)
Week 68	16	448.4 (147.35)	425.5 (260–701)	14	421.4 (180.35)	389.0 (141–782)

Abbreviations: ISL, islatravir.

Supplementary Table 6. Participants With AEs in the Infections and Infestations System Organ Class (Incidence >0% in Any Treatment Arm) by Percent Reduction From Baseline in Lymphocyte Count (10^9/L) of All Participants As Treated Through the End of the Active Treatment Period

	ISL 60 mg		ISL 120 mg		Placebo	
	≥30% reduction	<30% reduction	≥30% reduction	<30% reduction	≥30% reduction	<30% reduction
**n (%)**	**49**	**46**	**76**	**20**	**4**	**42**
**With ≥1 infections and infestations AEs**	**14 (28.6)**	**12 (26.1)**	**14 (18.4)**	**4 (20.0)**	**0**	**10 (23.8)**
Acrodermatitis	1 (2.0)	0	0	0	0	0
Bacterial vaginosis	0	0	1 (1.3)	0	0	0
Balanitis candida	0	0	0	0	0	1 (2.4)
Bronchitis	0	0	1 (1.3)	0	0	0
COVID-19	1 (2.0)	0	0	0	0	0
Conjunctivitis	0	0	1 (1.3)	0	0	0
Ear infection	0	0	1 (1.3)	0	0	0
Escherichia infection	0	0	1 (1.3)	0	0	0
Fungal foot infection	0	1 (2.2)	0	0	0	0
Fungal skin infection	1 (2.0)	1 (2.2)	0	0	0	0
Gastroenteritis	2 (4.1)	0	2 (2.6)	1 (5.0)	0	1 (2.4)
Gastroenteritis viral	1 (2.0)	0	1 (1.3)	0	0	0
Gingivitis	0	0	1 (1.3)	0	0	0
Hordeolum	1 (2.0)	0	0	0	0	0
Influenza	0	1 (2.2)	0	0	0	0
Ophthalmic herpes simplex	1 (2.0)	0	0	0	0	0
Oral herpes	1 (2.0)	0	0	0	0	0
Otitis media	1 (2.0)	0	1 (1.3)	0	0	0
Pharyngitis	0	1 (2.2)	0	0	0	0
Pharyngitis streptococcal	1 (2.0)	0	0	0	0	2 (4.8)
Sinusitis	0	0	0	1 (5.0)	0	0
Tinea versicolor	0	1 (2.2)	0	0	0	0
Tooth abscess	0	0	0	0	0	1 (2.4)
Tooth infection	0	1 (2.2)	0	0	0	0

	ISL 60 mg		ISL 120 mg		Placebo	
	**≥30% reduction**	**<30% reduction**	**≥30% reduction**	**<30% reduction**	**≥30% reduction**	**<30% reduction**
**n (%)**	**49**	**46**	**76**	**20**	**4**	**42**
Upper respiratory tract infection	3 (6.1)	1 (2.2)	3 (3.9)	1 (5.0)	0	3 (7.1)
Urethritis	0	0	1 (1.3)	0	0	0
Urinary tract infection	0	3 (6.5)	1 (1.3)	0	0	0
Viral infection	0	1 (2.2)	1 (1.3)	0	0	1 (2.4)
Viral upper respiratory tract infection	0	0	0	1 (5.0)	0	1 (2.4)
Vulvovaginal candidiasis	1 (2.0)	1 (2.2)	0	0	0	0
Vulvovaginal mycotic infection	1 (2.0)	0	0	1 (5.0)	0	1 (2.4)

Every participant is counted a single time for each applicable row and column. Participants with both baseline and ≥1 post-baseline lymphocyte test result were included.

Abbreviations: AEs, adverse events; COVID-10; coronavirus disease 2019; ISL, islatravir.

Supplementary Table 7. Summary Mean (%GCV) Pharmacokinetic Parameter Values of ISL in Plasma and ISL-TP in PBMC, Following Multiple Once-Monthly Administrations of Oral ISL in Participants at Low Risk of Acquiring HIV-1

Matrix	Analyte	PK Endpoint	ISL 60 mg				ISL 120 mg			
			Day 1		Week 20		Day 1		Week 20	
			n	GM (%GCV)	n	GM (%GCV)	n	GM (%GCV)	n	GM (%GCV)
**Plasma**	ISL[a]	$AUC_{0\text{-}672}$ (hr·µmol/L)	93	7.88 (56.8)	82	21.2 (140.3)	96	16.6 (50.5)	92	37.6 (136.6)
		C_{max} (µmol/L)	97	0.387 (279.9)	85	0.376 (597.7)	97	0.954 (186.2)	92	0.792 (349.6)
		C_{trough} (µmol/L)	92	0.000556 (36.6)[b]	85	0.000809 (37.6)[b]	95	0.00101 (37.0)[b]	92	0.00124 (42.0)
		T_{max}[c] (hr)	97	0.50 (0.42–24.05)	85	0.50 (0.00–188.77)	97	0.50 (0.38–25.97)	92	0.50 (0.37–311.73)
		T_{last}[c] (hr)	--	--	85	671.50 (0.00–1048.88)	--	--	92	671.55 (525.85–864.85)
		$t_{1/2}$ (hr)	--	--	81	175 (16.2)	--	--	92	177 (19.8)
**PBMC**	ISL-TP[d]	$AUC_{0\text{-}672}$ (hr·pmol/10^6 cells)	36	4720 (42.8)	37	2670 (167.8)	35	8490 (45.8)	38	4860 (50.0)
		C_{max} (pmol/10^6 cells)	37	20.5 (46.3)	37	9.15 (101.0)	37	41.4 (54.8)	38	17.1 (61.8)
		C_{trough} (pmol/10^6 cells)	36	0.975 (151.8)	35	1.69 (61.1)[b]	35	1.94 (65.5)[b]	37	1.96 (72.1)
		T_{max}[c] (hr)	37	24.20 (18.90–333.77)	37	167.75 (45.62–1341.78)	37	24.22 (17.00–212.57)	38	167.76 (0.00–672.23)
		T_{last}[c] (hr)	--	--	37	2688.12 (1341.78–4175.90)	--	--	38	2830.77 (1994.12–8063.23)
		$t_{1/2}$ (hr)	--	--	36	483 (36.1)	--	--	38	474 (34.9)

Abbreviations: $AUC_{0–672}$, area under the concentration-time curve from 0 to 672 hours; BLOQ, below the limit of quantitation; C_{max}, maximum concentration; C_{trough}, trough concentration; GM, geometric least-squares mean; GCV, geometric coefficient of variation; ISL, islatravir; PBMC, peripheral blood mononuclear cell; PK, pharmacokinetics; $t_{1/2}$, terminal elimination half-life; T_{last}, time to last measurable concentration; T_{max}, time to maximum concentration; TP,

triphosphate.

[a]1 participant in ISL 60 mg had all concentrations BLOQ at week 20, whereas 1 participant had insufficient data to calculate PK parameters at week 20. Hence, these 2 participants were excluded from the analysis.

[b]Values corresponds to arithmetic mean (%CV) as few subjects had C_{trough} as 0 or BLOQ. Thus, arithmetic mean (%CV) reported as geometric mean (%GCV) was not calculable.

[c]Median (Minimum – Maximum).

[d]2 participants in ISL 60 mg had measurable concentrations at day 1 predose and 1 participant in ISL 120 mg had insufficient data to calculate PK parameters on day 1. Hence, day 1 profiles of these participants were excluded from the analysis, whereas 1 participant in ISL 60 mg had all concentrations BLOQ at week 20 and 2 participants in ISL 120 mg had insufficient data to calculate PK parameters at week 20. Hence, week 20 profiles of these participants were excluded from the analysis.

Supplementary Figure 1. Concentration-time profiles of ISL or ISL-TP following multiple QM oral administrations of 60-mg and 120-mg doses in (*A* and *B*) plasma and (*C* and *D*) PBMCs, respectively.

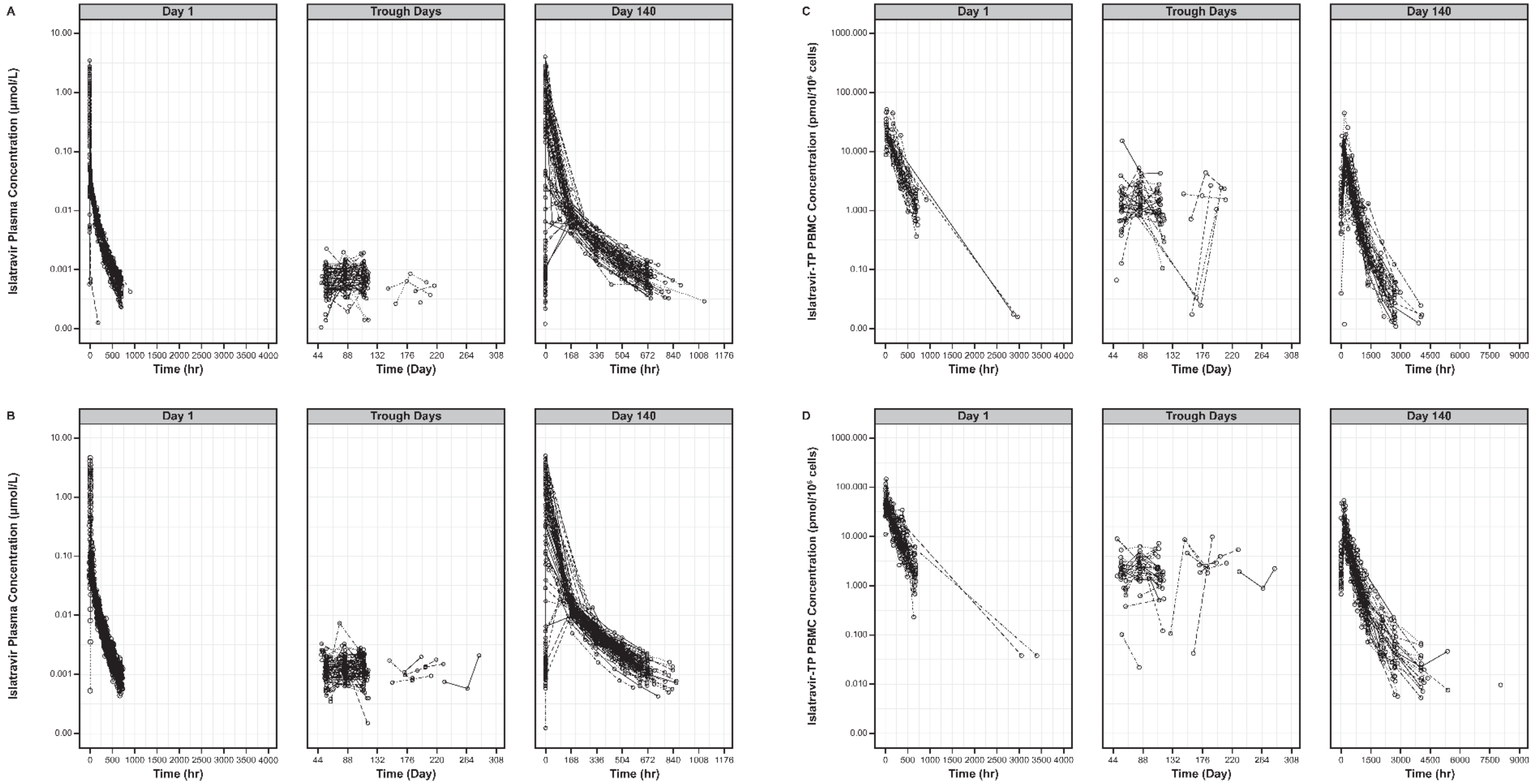

Abbreviations: ISL, islatravir; PBMC, peripheral blood mononuclear cell; PK, pharmacokinetics; QM, once monthly; TP, triphosphate.

1 participant in the ISL 60-mg group had all concentrations below the limit of quantitation at week 20, whereas 1 participant had insufficient data to calculate PK parameters at week 20. Hence, these 2 participants were excluded from the plasma analysis.

2 participants in 60-mg dose group had measurable concentrations at day 1 predose, whereas 1 participant in 60-mg dose group had all concentrations below the limit of quantitation at week 20. Hence, these participants were excluded from the PBMC analysis.

Trough days correspond to actual time in days for weeks 8, 12, and 16 predose.
